# Occurrence and risks of pharmaceuticals in Mahdia’s coastline (Tunisia): distribution, antibiotic resistance, and ecotoxicological impact

**DOI:** 10.1007/s11356-025-36743-3

**Published:** 2025-07-24

**Authors:** Ferdaws Fenni, Adrià Sunyer-Caldú, Hedi Ben Mansour, Maria Silvia Diaz-Cruz

**Affiliations:** 1 Research Unit of Analysis and Procedures Applied to the Environment-APAE UR17ES32, University of Tunis El Manar, Tunis, 1068 Tunisia; 2 Department of Environmental Science (ACES, Exposure & Effects), Science for Life Laboratory, Stockholm University, Stockholm, 106 91 Sweden; 3 Consejo Superior de Investigaciones Cientificas (CSIC), Institute of Environmental Assessment and Water Research (IDAEA), Jordi Girona 18, Barcelona, 08034 Spain

**Keywords:** Antibiotics resistance, PHACs, Wastewater, Seawater, Environmental risk, Marine pollution

## Abstract

Pharmaceutical active compounds (PHACs) pose a significant risk to aquatic ecosystems due to their persistence and potential toxic effects. Despite their documented presence in various coastal environments, their occurrence along the Mahdia coastline remains largely unexplored. Using ultra-performance liquid chromatography-tandem mass spectrometry (UHPLC-MS/MS), we analyzed seawater and sediment samples collected over six consecutive months from Mahdia’s marine environment. Results revealed that antibiotics were the most prevalent PHACs, with ofloxacin reaching 401 ng/L in seawater. Other consistently detected compounds included flumequine, nalidixic acid, oxytetracycline, and N4-acetylsulfamerazine, with maximum concentrations of 16 ng/L, 17.1 ng/L, 12.1 ng/L, and 59.2 ng/L, respectively. Additionally, carbamazepine was detected at 5.2 ng/L, while caffeine concentrations ranged from 12.6 ng/L to 638 ng/L in seawater and up to 6.87 ng/g in sediment. The study also assessed antibiotic resistance risks, expressed as resistance risk quotients (RRQs) and indicating that ofloxacin and flumequine pose a medium-risk threat to bacterial communities. Ecotoxicological evaluations through calculation of ecotoxicological risk quotient (ERQs) and hazard indexes (HI) classified ofloxacin as a low-to-moderate risk for fish populations. These findings provide the first comprehensive mapping of PHAC contamination and potential risks along the Mahdia coast, emphasizing the urgent need for improved wastewater treatment and monitoring programs to mitigate environmental risks.

**Graphical Abstract:**

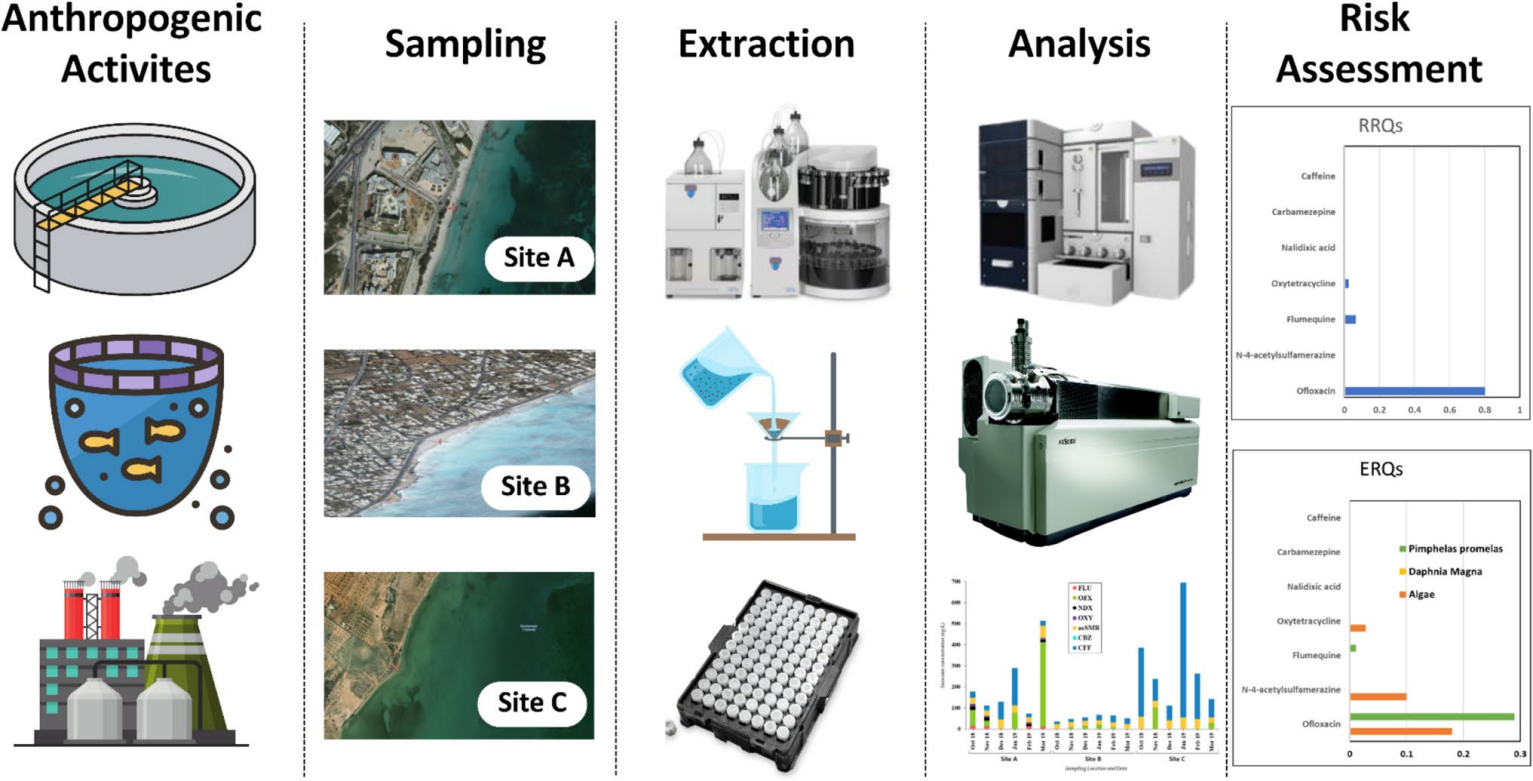

**Supplementary Information:**

The online version contains supplementary material available at 10.1007/s11356-025-36743-3.

## Introduction

Pharmaceutical active compounds (PHACs) are widely used in human and veterinary medicine, improving global health. However, their continuous discharge into the environment has led to their accumulation in aquatic ecosystems, primarily due to inefficient removal by conventional wastewater treatment plants (WWTPs) (Telgmann and Horn 2024). This issue is particularly concerning in developing countries, where wastewater treatment facilities often lack advanced purification processes (Bolesta et al. 2022).

PHACs are considered pseudo-persistent contaminants due to their continuous input and potential bioaccumulation (De Jesus Gaffney et al. 2017). While their presence in freshwater systems has been well-documented, their distribution in marine environments remains less studied, mainly due to analytical challenges related to their low concentrations and complex seawater matrices (Gaw et al. 2014). Recent advances in ultra-trace detection techniques have improved the identification and quantification of PHACs in seawater (Sang et al. 2022), enabling a more comprehensive assessment of their occurrence and impacts.

Among PHACs, antibiotics are of particular concern due to their role in antimicrobial resistance (AMR) development. Their release into marine environments is exacerbated by hospital effluents, aquaculture, and urban wastewater discharges (Ahmed et al. 2024). In Tunisia, antibiotic consumption exceeds 45 million defined daily doses (DDD) per 1000 inhabitants, positioning the country among the highest global users (Klein et al. 2024). The Mahdia governorate, a key economic region in Tunisia, relies on fishing, aquaculture, and tourism (Fenni et al. 2022; Hzmdi et al. 2019). Aquaculture is particularly prominent, with the intensive farming of species such as sea bass (*Dicentrarchus labrax*) and sea bream (*Sparus aurata*) for both local consumption and export (Abdou et al. 2018). While some studies have reported the occurrence of antibiotic residues in pharmaceutical industry (Tahrani et al. 2018) and urban wastewaters (Harrabi et al. 2018), these efforts have largely focused on effluent sources, with little attention paid to their fate and distribution in the receiving marine environment.

In this context, the present study pursues to fill this gap by providing a comprehensive assessment of pharmaceuticals’ contamination along the Mahdia coastline. Specifically, we investigated the occurrence, spatial variability, and environmental risks of selected PHACs, including antibiotics, in both seawater and sediment across three coastal sites with distinct anthropogenic pressures. Our aim was to evaluate whether differences in local wastewater inputs, proximity to aquaculture facilities, and WWTP discharge points translate into divergent contaminant profiles and risk levels in the receiving marine environment. By calculating environmental risk quotients (ERQs) based on measured environmental concentrations and established predicted no-effect concentrations (PNECs), we also evaluated the likelihood of adverse ecological effects.

We hypothesize that site-specific human activities drive the spatial variability of pharmaceutical contamination, and that sediment matrices due to their role as sinks for hydrophobic and particulate-bound contaminants, may serve as long-term indicators of pollutants exposure. Through this study, we aim to not only establish a robust baseline for pharmaceutical pollution in this understudied coastal region but also to contribute to broader discussions on marine ecotoxicology, antimicrobial resistance risks, and the effectiveness of current wastewater management practices in North African contexts.

## Materials and methods

### Sampling sites and sample collection

**Fig. 1:**
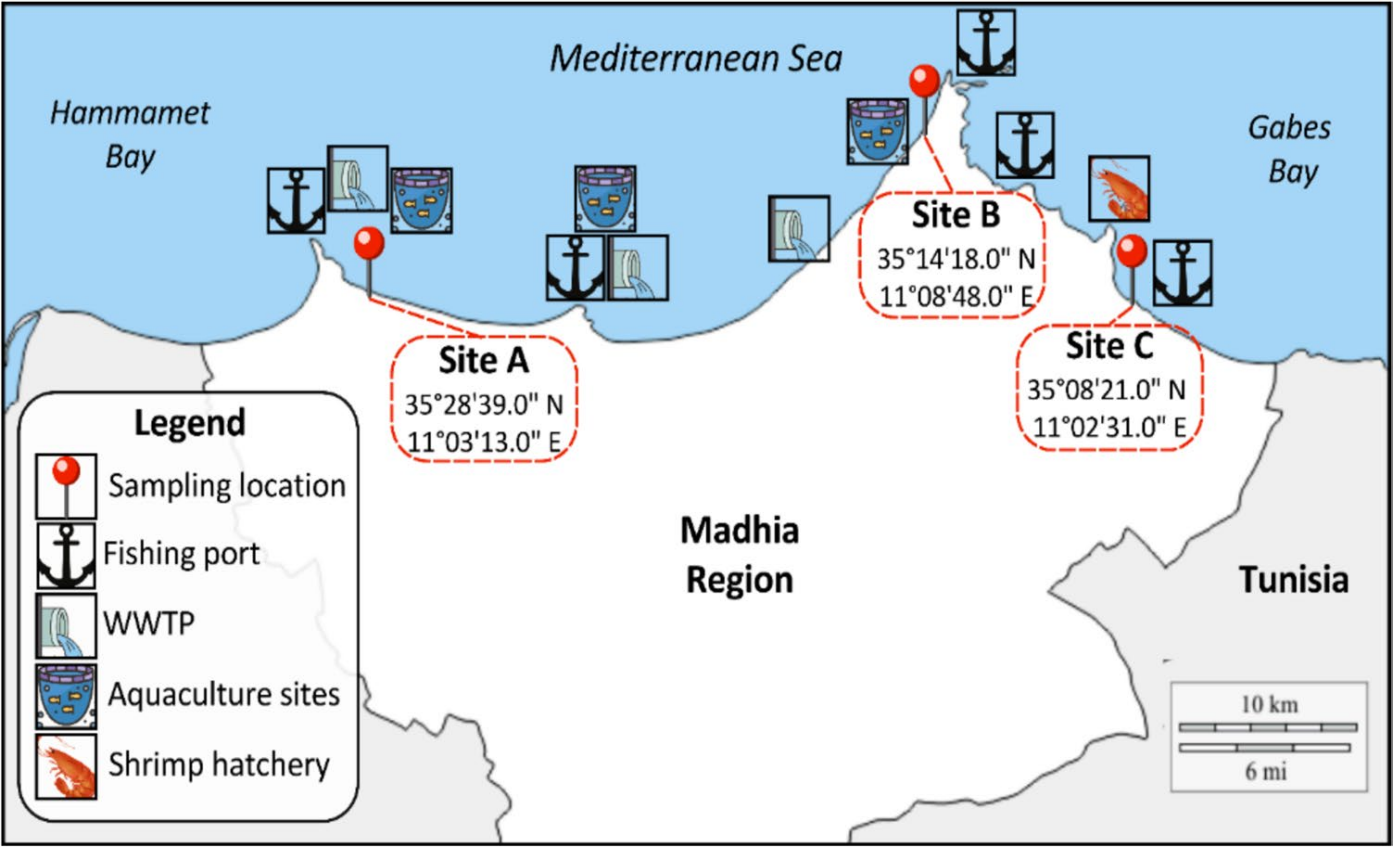
Sampling locations and nearby anthropogenic activities along the Mahdia coastline (Tunisia)

Three coastal sites along the Mahdia shoreline in Tunisia were selected based on their varying extent of anthropogenic influence and environmental conditions (Fig. 1). Site A (35°28′39″N; 11°03′12″E) is a sandy beach located 830 m from a wastewater treatment plant (WWTP) discharge point, adjacent to seabream aquaculture cages and a fishing port, and commonly frequented by tourists. Site B (35°14′18″N; 11°08′44″E), situated within Hammamet Bay, is a large recreational beach with nearby aquaculture activities and located 6.2 km downstream from the same WWTP. Site C (35°08′21″N; 11°02′31″E), in Gabes Bay, is characterized by high tidal amplitude, clayey sediments, and shallow waters. It is influenced by nutrient inputs contributing to eutrophication and lies near a shrimp hatchery (4.6 km upstream) and a small fishing port (1.5 km north).

Sampling campaigns were conducted from October 2018 to March 2019. At each site, seawater and sediment samples were collected following a composite sampling approach to ensure spatial representativity. For seawater, 1 L was collected from five points arranged in an X-pattern at each of three locations spaced 200 m apart (total of 15 points per site), 10 m from the shore and at 50 cm depth. Samples were combined into a single composite sample per site per campaign and stored in sterile brown glass bottles.

For sediment, samples were collected at the same three locations per site (200 m apart) using a Van Veen grab sampler, targeting the top 0–10 cm layer over 0.1 m^2^. Five replicate grabs were collected per location, resulting in 15 subsamples per site. Sediments were placed in sterile glass containers, transported in a cooler, and pooled in the lab to form a representative composite per site. This strategy ensured a robust dataset while minimizing local variability.

### Standards and reagents

A comprehensive list of PHACs was selected based on detection frequency in previous studies and toxicity potential (Afsa et al. 2020; Fenni et al. 2022; Jebara et al. 2021; Sunyer-Caldú et al. 2023). Selected PHACs physicochemical properties are listed in Table 1. A detailed description of the analytical standards, reagents, preparation of stock solutions, and commercial suppliers can be found in Section S1 of the SI.

**Table 1:** Main physicochemical properties of the investigated compounds. S, water solubility at 25 °C

Compound	Acronym	Molecular formula	CAS#^a^	pKa^a^	LogK_ow_^a^	S^a^
Flumequine	FLU	C_14_H_12_FNO_3_	42835–25-6	6.5	1.6	Insoluble
Ofloxacin	OFX	C_18_H_20_FN_3_O_4_	82419–36-1	5.97	− 0.39	28.3 mg/mL
Ciprofloxacin	CFX	C_17_H_18_FN_3_O_3_	85721–33-1	6.09	0.28	< 1 mg/mL
Nalidixic acid	NDX	C_12_H_12_N_2_O_3_	389–08-2	8.6	1.41	Insoluble
Oxalinic acid	OXL	C_13_H_11_NO_5_	14698–29-4	6.3	1.7	3.21 mg/mL
Tetracycline	TCY	C_22_H_24_N_2_O_8_	60–54-8	3.3	− 1.37	231 mg/L
Oxytetracycline	OXY	C_22_H_24_N_2_O_9_	79–57-2	3.27	− 0.90	313 mg/L
Succinyl sulfathiazole	SSTZ	C_13_H_13_N_3_O_5_S_2_	116–43-8	4.6	0.05	< 1 mg/mL
Sulfadiazine	SDZ	C_10_H_10_N_4_O_2_S	68–35-9	6.36	− 0.09	77 mg/L
N4-acetylSulfadiazidine	acSDZ	C_12_H_12_N_4_O_3_S	127–74-2	6.08	− 0.2	0.5 mg/mL
Sulfamerazine	SMR	C_11_H_12_N_4_O_2_S	127–79-7	0.14	0.14	202 mg/L
N4-acetylSulfamerazine	Ac-SMR	C_13_H_14_N_4_O_3_S	127–73-1	-	-	< 1 mg/mL
N4-acetylSulfamethiazine	acSMZ	C_14_H_16_N_4_O_3_S	100–90-3	-	-	-
Sulfamethoxazole	SMX	C_10_H_16_N_4_O_3_S	723–46-6	5.7	0.89	< 1 mg/mL
N4-acetylSulfamethoxazole	acSMX	C_12_H_13_N_3_O_4_	21312–10-7	-	0.7	< 1 mg/mL
Sulfamethoxypyridazine	SMPZ	C_11_H_12_N_4_O_3_S	80–35-3	-	-	< 1 mg/mL
Sulfapyridine	SPY	C_11_H_11_N_3_O_2_S	144–83-2	8.43	0.35	268 mg/L
N4-Sulfapyridine	acSPY	C_13_H_13_N_3_O_3_S	19077–98-6	-	− 0.01	-
Sulfaquinoxaline	SQX	C_14_H_12_N_4_O_2_S	59–40-5	5.1	1.68	< 1 mg/mL
Sulfathiazole	STZ	C_9_H_3_N_3_O_2_S_2_	72–14-0	7.2	0.05	373 mg/L
Sulfisomidine	SMD	C_12_H_14_N_4_O_2_S	515–64-0	7.25	1.2	-
Sulfadimethoxine	SDM	C_12_H_14_N_4_O_4_S	122–11-2	5.94	1.63	343 mg/L
Trimethoprim	TMP	C_15_H_22_O_3_	738–70-5	7.12	0.91	400 mg/L
Gemfibrozil	GFZ	C_15_H_15_NO_2_	25812–30-0	4.5	4.8	27 mg/L
Mefamicacid	MFA	C_14_H_14_O_3_	61–68-7	4.2	5.12	20 mg/L
Naproxen	NPX	C_13_H_18_O_2_	22204–53-1	4.15	3.18	15.9 mg/L
Iburofen	IBU	C_16_H_14_O_3_	15687–27-1	5.3	3.97	21 mg/L
Ketoprofen	KPF	C_16_H_14_O_3_	22071–15-4	3.12	4.45	51 mg/L
Diclofenac	DCF	C_14_H_11_C_l2_NO_2_	15307–86-5	4.15	4.51	2.37 mg/L
Paracetamol	APH	C_8_H_9_NO_2_	103–90-2	9.5	0.46	14 g/L
Carbamazepine	CBZ	C_15_H_12_N_2_O	298–46-4	13.9	2.45	< 1 mg/mL
Epoxycarbamazepine-10,11	CBZ-E	C_15_H_12_N_2_O_2_	36507–30-9	-	-	-
Atenol	ATL	C_14_H_22_N_2_O_3_	29122–68-7	9.6	0.16	13.3 g/L
Nofloxetine	NorFXT	C_16_H_16_F_3_NO	83891–03-6	9.05	3.5	< 1 mg/mL
N-Desmethylvenlafaxine	N-desVFX	C_16_H_25_NO_2_	142761–12-4	-	2.6	-
Caffeine	CFF	C_8_H_10_N_4_O_2_	58–08-2	14	− 0.07	21.6 mg/mL

^a^From the U.S. National Library of Medicine (2025)

### Physicochemical characterization of seawater samples

Upon arrival at the laboratory, the homogenized seawater samples were analyzed for various parameters. Biochemical oxygen demand (BOD), chemical oxygen demand (COD), total organic carbon (TOC), total suspended solids (TSS), and nitrate concentration (NO₃⁻) were measured using a portable ultraviolet (UV) analyzer (Pastel UV, Secomam, Alès, France). Electrical conductivity (EC) and pH were determined with a WTW 315i conductometer and a WTW pH meter (Weilheim, Baviera, Germany) respectively.

### Biodegradability ratio

To assess the potential of organic compounds in seawater to be broken down by microbial activity the degradability ratio was determined using Eq. 1. A higher ratio indicates that the organic matter is more readily biodegradable, while a lower ratio suggests that the compounds are more resistant to degradation. Biodegradability ratio was:

1 $$BOD/{COD}_{Ratio}=BOD/COD$$

BOD and COD were determined separately for each seawater sample. The BOD/COD ratio was then calculated for each sample individually. A DBO/DCO ratio close to 1 suggests that a significant portion of the organic matter is biodegradable. Ratios lower than 1 indicate that a substantial portion of the organic matter is non-biodegradable or toxic, potentially affecting water quality (Samudro, and Mangkoedihardjo 2010).

### Seawater and sediment sample treatment

Water and sediment were pre-treated and extracted following previously published protocols (Gago-Ferrero et al. 2011). Seawater samples (0.5 L) were filtered through 0.45-µm nylon and 0.2-µm glass fiber filters before analysis. Sediment samples were homogenized and freeze-dried. A 1 g portion of sediment was spiked-covered with the solution of the surrogate standards and acetone (100 µL BP-13C; 1 mg/L) before extraction by pressurized liquid extraction (ASE-350, Dionex). The extraction process involved two cycles: the first using as extraction solvent 10 mL of methanol (MeOH) and the second using 10 mL of a MeOH:water mixture (50:50, v:v). The extracts were combined and brought to a final volume of 25 mL with MeOH. An aliquot of 2 mL from this solution was filtered into an LC vial. After evaporating the filtrate to near dryness under nitrogen, 50 µL of an internal standard mix solution (1 µg/mL) was added, and MeOH was added up to 1 mL, and then stored at − 20 °C until analysis.

### Chemical analysis

Instrumental analysis was conducted using ultra-high performance liquid chromatography-tandem mass spectrometry (UHPLC-MS/MS), following the protocols from our previous studies (Soares et al. 2021).

A brief description is provided below:

#### Seawater samples

Seawater samples were pre-concentrated, extracted, and purified through on-line solid-phase extraction (on-line SPE) in a Symbiosis TM Pico system from Spark Holland (Emmen, The Netherlands). During the on-line SPE, 5 mL of samples, aqueous standard solutions, and blanks was loaded at a flow rate of 1 mL/min through a pre-conditioned PLRP-s cartridge. The cartridge was conditioned with 1 mL of methanol (MeOH), 1 mL of acetonitrile (ACN), and 1 mL of HPLC water. Following sample loading, cartridges were washed with HPLC water, followed by elution using the chromatographic mobile phase, which was directed through the SPE column. Target analytes were separated using a Hiber Purospher®STAR® HR R-18ec (50 × 2.0 mm, 2 µm) LC-column provided by Merck, on an Acquity UHPLC chromatograph (Waters, Milford, MA, USA). In all analyses, a 20 µl injection volume and a mobile phase flow rate of 0.3 mL/min were employed. Detection was conducted using a 4000 QTRAP MS/MS analyzer from Applied Biosystems-Sciex (Foster City, CA, USA) in selected reaction monitoring (SRM) mode. Electrospray ionization (ESI) was the selected ionization technique, operating in both positive and negative modes. Further information about the methodology can be found in Tables S1 to S4.

#### Sediment samples

The HPLC–MS/MS conditions for sediment analysis were consistent with those used for water samples, but the sample extraction was performed off-line. Additional information about the methodology is provided in Tables S1 to S4.

#### QA/QC

The identification of all compounds relied on chromatographic retention time (RT) and the two most intense SRM transitions (first for quantification and second for confirmation, Table S3), following the guidelines outlined by the European Commission (96/23/EC, 1996). Working materials were meticulously cleaned with ethanol and acetone. Non-volumetric glassware was muffled at 400 °C. Procedural blanks and quality control samples were randomly introduced into the analysis sequence every ten samples to ensure analytical accuracy. The limits of detection (LOD) and quantification (LOQ) were determined based on the signal-to-noise (S/N) ratio obtained from the mass spectrometric data. Specifically, the LOD was defined as the lowest concentration of each compound yielding an S/N ratio of at least 3, while the LOQ corresponded to the concentration at which the S/N ratio reached or exceeded 10. These values were calculated by analyzing low-concentration standards and measuring the ratio of the analyte signal to the baseline noise within the corresponding chromatographic window. Accuracy was evaluated based on the recovery rate of each standard spiked into blank water or sediment samples, analyzed in triplicate at two concentration levels (5 × LOQ, and 10 × LOQ). Precision was expressed as the relative standard deviation (RSD, %) for each concentration level, assessed intra-day. These data are provided in Table S4.

### Environmental risk assessment

To evaluate ecotoxicological risk, two risk hazards estimates were calculated: the resistance risk quotient (RRQ) and the ecotoxicological risk quotient (ERQ).

#### Resistance risk quotient

The resistance risk quotient is a specific application of the risk quotient (RQ), defined as the ratio of the predicted environmental concentration (PEC) to the predicted no effect concentration for resistance (PNEC-R). PNEC-R represents the threshold concentration below which antimicrobial resistance is not expected to be promoted and is typically derived from minimum inhibitory concentration (MIC) data. The RRQ focuses on the environmental impact of antimicrobial agents and their potential to promote resistance in microbial populations.

It is calculated using Eq. 2:

2 $$RRQ=\frac{PEC}{PNEC-R}$$

In this study, measured environmental concentrations were used as PEC values for risk assessment purposes. PNEC-R values were retrieved from the NORMAN Ecotoxicology database (antimicrobial resistance only) (DuLio et al. 2020). An RRQ greater than 1 indicates a potential risk for selecting antimicrobial resistance, while values below 1 suggest minimal risk.

#### Ecotoxicological risk quotient

The evaluation of ecotoxicological hazards associated with PHACs is a crucial process for assessing the potential adverse effects of chemicals on ecosystems. This assessment plays a key role in environmental protection and management (Gosset et al. 2021). As part of this evaluation, we first determined the estimated environmental concentration (EEC), which represents the maximum measured concentration of each contaminant in our samples. This value was then divided by the predicted no effect concentration (PNEC), a toxicity threshold obtained from the NORMAN database (DuLio et al. 2020). Based on this approach, the ERQ was calculated using Eq. 3:

3 $$ERQ= \frac{EEC}{PNEC}$$

The cumulative risk posed by the detected contaminants at each sampling site was estimated based on the additivity model and expressed as hazard index (HI), which was calculated using Eq. 4:

4 $$HI= \sum \frac{Ci}{PNECi} = {\sum }ERQs$$

PNEC values used for this calculation were obtained from the NORMAN database (DuLio et al. 2020) and calculated based on exposure to the fish species *Pimephales promelas*.

### Data analysis

Statistical analyses were conducted using SPSS 15.0 for Windows (SPSS Inc., Chicago, IL, USA). The normality of the data was assessed using the Shapiro–Wilk test, while Levene’s test was employed to evaluate the homogeneity of variances. Averages were compared through ANOVA, and the differences between means were analyzed using either Tukey’s HSD or T2 tests, depending on the homogeneity of variances. The relationships and co-variability among contaminants and other variables were examined using linear correlation (Pearson) and factorial analysis. A significance level of 5% was established for all statistical analyses.

## Results and discussion

### Physicochemical properties of seawater samples

All measured parameters remained below the maximum allowed concentrations set by Tunisian regulations (Tunisian Institute for Standardization 1989). However, the relatively higher pollutant levels at Site C suggest localized contamination sources (Table 2).

**Table 2:** Physicochemical data for seawater samples collected over a 6-month campaign (data expressed as mean values with confidence intervals)

Parameters	Maximum allowed concentrations^a^	Site A	Site B	Site C
pH	6.5 < pH < 8.5	7.58 ± 0.63	7.80 ± 0.28	7.94 ± 0.25
COD (mg/L)	90 mg O_2_/L average 24 h	6.64 ± 1.17	6.89 ± 1.48	10.56 ± 1.62*
BOD (mg/L)	30 mg O_2_/L average 24 h	4.14 ± 1.99	4.45 ± 1.97	5.69 ± 1.32
BOD/COD	-	0.53 ± 0.20	0.78 ± 0.20	0.46 ± 0.20*
TOC (mg/L)	30	2.37 ± 1.02	1.48 ± 0.22	3.02 ± 1.14
NO_3_^−^ (mg/L)	90	4.61 ± 0.43	4.98 ± 0.32	5.55 ± 0.55
EC (µS/cm)	50,000	27,076 ± 2,778	27,276 ± 4,461	39,644 ± 6,537*
TSS (mg/L)	35	11.37 ± 1.07	11.96 ± 1.41	21.17 ± 7.99*

*COD* chemical oxygen demand, *BOD* biochemical oxygen demand, *TOC* total organic carbon, *NO*_*3*_^*−*^ nitrate, *EC* electrical conductivity, *TSS* total suspended solids, *µS/cm* microsiemens per centimeter

^a^(Tunisian Institute for Standardization 1989) Tunisian Norm relating to discharges of effluents into the aquatic environment

^*^Statistically significant differences (*p* ≤ 0.05) compared to those values for site A and B according to Tukey’s HSD test

The physicochemical properties at site C were higher than at sites A and B, with significant increases in COD, EC, and TSS (*p* ≤ 0.05), indicating a greater presence of organic matter and potential pollution. In contrast, no statistically significant differences were observed in TOC, BOD, pH, or NO3^−^ levels across the sites (*p* ≥ 0.05). The notably higher electrical conductivity (EC) at site C, with values of 39,644 ± 6,537 µS/cm, suggests elevated salinity or the presence of dissolved inorganic pollutants, likely from industrial discharges, agricultural runoff, or wastewater effluents. The increased TSS concentration at site C (21.17 ± 7.99 mg/L) points to a higher potential for particle-associated contaminants, such as hydrophobic organic pollutants and nutrients, which may lead to sediment deposition and long-term environmental impacts. The biodegradability ratio, defined as the ratio of BOD to COD, is commonly used to evaluate microbial degradation capacity (Jiao et al. 2021). Site B showed the highest BOD/COD ratio (0.78 ± 0.20), indicating more biodegradable organic matter, while site C had the lowest ratio (0.46 ± 0.20).

The low biodegradability ratio at site C’s seawater samples can be attributed to a combination of environmental and anthropogenic factors. In shallow waters with clay sediments, organic matter binds tightly to clay particles, limiting its accessibility to microorganisms (Diwyanjalee et al. 2024). High tides and weak currents further restrict water dispersion and oxygenation, hindering aerobic biodegradation (Béjaoui et al. 2019). Moreover, the unconventional discharge of waste, including synthetic chemicals and heavy metals (Fenni et al. 2022), introduces recalcitrant compounds that resist microbial breakdown, further lowering the biodegradability ratio. These factors together create an environment where organic pollutants persist longer, diminishing the biodegradation capacity of seawater.

### Occurrence and spatial distribution of PHACs

#### Seawater

**Fig. 2:**
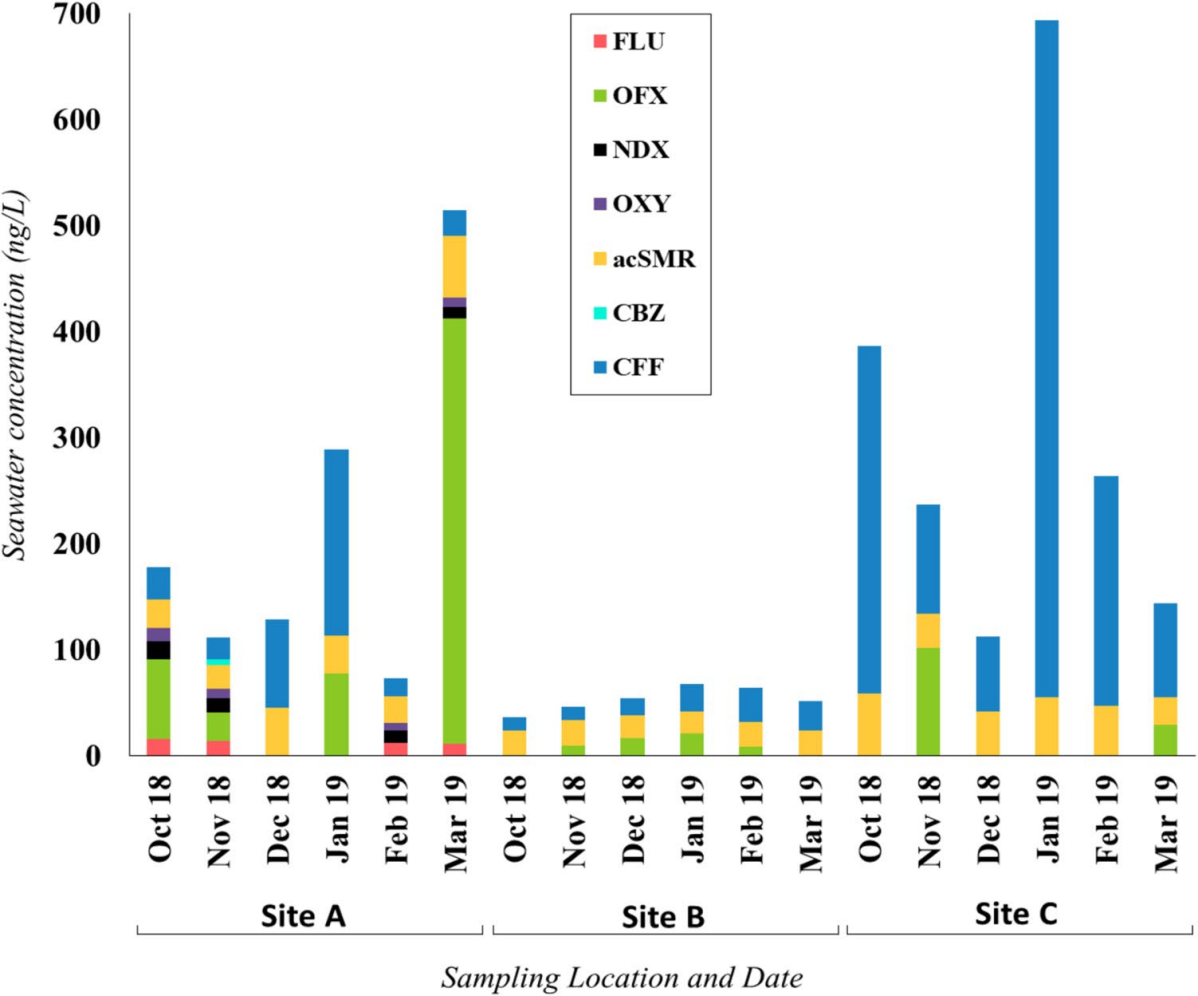
Spatial and temporal distribution of PHACs in seawater across Mahdia shoreline. FLU, flumequine; OFX, ofloxacin; NDX, nalidixic acid; OXY, oxytetracycline; acSMR, N^4^-acetylsulfamerazine; CBZ, carbamazepine; CFF, caffeine

Chemical analysis targeted thirty-seven compounds across eight pharmaceutical classes, including twenty-three antibiotics, one lipid regulator, six anti-inflammatory agents, one analgesic, two antiepileptics, one β-blocker, two antidepressants, and one stimulant (Table S3). Antibiotics were the predominant PHACs in seawater, accounting for 73% of the total detected PHACs. Antibiotics were found in 43.3% of the seawater samples, while the stimulant CFF was present in all samples, and CBZ was detected only once in site A at a concentration of 5.2 ng/L (Table 3). Notably, the antibiotics ofloxacin (OFX), flumequine (FLU), oxytetracycline (OXY), nalidixic acid (NDX), and N4-acetyl-sulfamerazine (ac-SMR) were determined at the highest concentrations (Fig. 2).

**Table 3:**
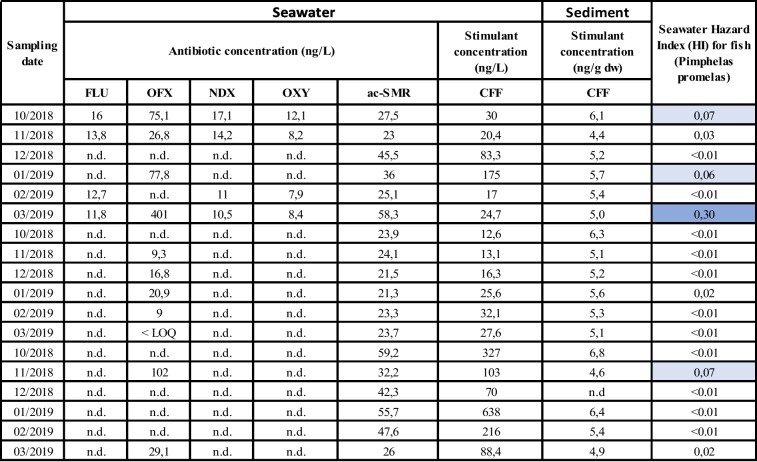
Spatial and temporal occurrence of PHACs in seawater and sediment along the shoreline of Mahdia and estimated hazard indexes (HI)

*FLU* flumequine, *OFX* ofloxacin, *NDX* nalidixic acid, *OXY* oxytetracycline, *ac-SMR* N^4^-cetylsulfamerazine, *CBZ* carbamazepine, *CFF* caffeine, *n.d.* not detected, < *LOQ* below limit of quantification

##### Antibiotics

Our study provides the first evidence of OFX occurrence in Tunisian seawater. OFX is a fluoroquinolone antibiotic commonly used to treat urinary and digestive infections in humans, but also reported to be effective against both Gram-negative and Gram-positive bacteria (Cao et al. 2021). OFX was detected in all seawater samples, with concentrations ranging from 26.8 to 401 ng/L at site A, 29.1 to 102 ng/L at site C, and 9 to 20.9 ng/L at site B. The spatial distribution suggests that its presence at site A is associated with wastewater discharges near the sampling point (Fig. 2), including untreated effluents from hospitals and poultry processing facilities. Additionally, seabream aquaculture cages are visibly installed along the shoreline in this area, potentially contributing to the contamination. At site C, the main source of OFX is likely the shrimp hatchery located 4.6 km upstream (Bermdez-Almada and Espinosa-Plascenci 2012) which lacks proper waste treatment and discharges directly into seawater. Compared to previous studies, OFX concentrations in Tunisian seawater are considerably higher than those reported in wastewater from pharmaceutical industries (0.3–3.1 ng/L) (Tahrani et al. 2018) but lower than levels found in hospital effluents, such as those from Mahdia Hospital (Tunisia), where mean concentrations reached 78,850 ng/L (Nasri et al. 2023).

Although OFX is susceptible to photodegradation, it can persist in seawater for up to 21 days (Hagenbuch & Pinckney 2012; Liu et al. 2023). Its presence in aquatic environments is primarily due to inefficient removal during wastewater treatment, allowing its accumulation in marine ecosystems. Conventional treatment plants often fail to fully eliminate OFX, leading to its continuous release into the environment. This raises concerns about long-term ecological impacts, including the emergence of antibiotic-resistant bacteria and disruptions in aquatic food webs.

Sulfonamides are widely used antibiotics in veterinary practices for treating diseases in poultry, cattle, and fish (EU Commission 2015; Lajnef et al. 2024). In our study, we analyzed 15 sulfonamide compounds and metabolites. The ac-SMR, major human metabolite of SMR, was found in all samples. Ac-SMR is commonly prescribed to treat bronchitis, prostatitis, and urinary tract infections (Djoumbou-Feunang et al. 2019). On average, ac-SMR was found at 43.8 ng/L in site C, 35.9 ng/L in site A, and 22.96 ng/L in site B. Its dissemination is closely related to WWTP effluents. Previous studies have shown that ac-SMR and acetyl-sulfamethoxazole in human excretion account for over 50% of the administered dose and can be present in WWTP influents at concentrations 2.5 to 3.5 times higher than those of the parent compound (Le-Minh et al. 2010; Tong et al. 2019). In site C, the highest concentration of ac-SMR can be attributed to the degradation of SMR, favored by the high levels of TSS (Tang et al. 2021). Occurrence of SMR at this site can be attributed to the nearby shrimp hatchery, as previously discussed for OFX (Hu et al. 2019). In site A, the proximity of the WWTP discharge to the sampling point, less than 1 km, potentially reduced the seawater dilution factor resulting in higher concentrations compared to site B, where the distance from the WWTP is about 6.2 km (Szopińska et al. 2022). In a previous study in coastal waters from site A, 6 sulfonamides, namely sulfadiazine (6–11 ng/L), sulfamethoxazole (2–6 ng/L), sulfathiazole (n.d–3 ng/L), sulfamethazole (4–11 ng/L), sulfamethazine (n.d–3 ng/L), and sulfamethoxy pyridazine (n.d–5 ng/L), were reported (Afsa et al. 2020). Another study across 5 coastal cities in Tunisia reported a continuous release of sulfamerazine into seawater at a mean concentration of 4.5 µg/L (Tahrani et al. 2018). This work, therefore, provides the first documented data of ac-SMR presence in seawater in Tunisia.

Three of the target antibiotics, namely flumequine (FLU), oxytetracycline (OXY), and nalidixic acid (NDX), were exclusively detected at site A. FLU is a fluoroquinolone primarily used in human medicine for treating urinary tract infections. However, in countries like Tunisia, FLU is also utilized to manage digestive infections caused by *Escherichia coli* and *Salmonella* (Majalekar & Shirote 2020). It is also used in veterinary medicine as preventive agent for enteric infections in food animals and bacterial infections in farmed fish. FLU was exclusively detected in 67% of the seawater samples from site A, with an average concentration of 16 ng/L. Similar to many other antibiotics, FLU’s presence in seawater is likely associated with nearby WWTP discharges and the presence of aquaculture cages located within 2 km of the sampling site. The measured FLU concentrations were very similar to the levels reported in Chilean Patagonia (12 ng/L). To date, its occurrence in Tunisia has been reported in WWTP effluents of Sfax (175.01 ng/L) (Harrabi et al. 2018) and in pharmaceutical industry waste effluents in Tunis (9.7 ng/L) (Tahrani et al. 2018). Transformation pathways of FLU in seawater via hydroxylation, decarboxylation, and defluorination have the potential to generate at least 13 transformation products (Xiao et al. 2023), underscoring the importance of assessing the occurrence of these derivatives alongside the parent compound for a comprehensive environmental assessment. OXY is a tetracycline antibiotic frequently used in both human and veterinary medicine. It is used to treat infections of the respiratory and urinary tracts (Fernandes et al. 2020). Besides, OXY is commonly used in fish hatcheries and aquatic farming in Tunisia (Lajnef et al. 2024). The presence of OXY was observed in 67% of the samples from site A. Mean concentrations ranged from 7.9 to 12.1 ng/L. This concentrations are of the same order of the reported mean concentration in coastal waters of the Gulf of Cadiz (Spain), 25 ng/L (Biel-Maeso et al. 2018). OXY is usually administered both orally and into the feed of seabream and has a low bioavailability; thus, it is quickly excreted into seawater without undergoing significant metabolism within the fish (Agwuh 2006). Similarly, OXY occurrence in site A is probably related to the closely installed bream cages. NDX is a quinolone antibiotic found exclusively at site A, with concentrations ranging from 1.89 to 17.1 ng/L. NDX is a first-generation quinolone drug used to treat urinary tract infections caused by susceptible Gram-negative microorganisms, including most *E. coli*, *Enterobacter*, *Klebsiella*, and *Proteus* species. The observed concentrations are lower than those detected in surface water from the Inanda Dam in South Africa, which reached 2.53 µg/L (Jiao et al. 2021). To the best of author’s knowledge, this study represents the first determination of FLU, OXY, and NDX in Tunisian seawater.

##### Other PHACs

Among the PHACs, CFF was the most frequently detected stimulant, present in all seawater samples across the investigated sites. Its highest concentration of 638 ng/L was recorded at site C, followed by 175 ng/L at site A and 32.1 ng/L at site B (Table 3). Despite its high biodegradability (Muter et al. 2017), the widespread presence of CFF in open seawater is likely linked to its extensive consumption in coffee and energy drinks, leading to its release through WWTP effluents (Li et al. 2020; Raj et al. 2021). Additionally, its use in pharmaceutical formulations, particularly analgesics designed to prolong therapeutic effects, represents another significant source of contamination (Nunes et al. 2022). As a pollutant, CFF stands out as one of the most prevalent in the environment serving as an indicator of anthropogenic input of PHACs into seawater (Grujić-Letić et al. 2016). The notably high CFF concentration at site C may be attributed to the area’s environmental characteristics. Its extensive continental shelf creates shallow, sheltered waters with limited hydrodynamic activity, favoring pollutant accumulation (Hattour et al. 2010). Similar studies in the Mediterranean have reported lower CFF concentrations, such as 78.2 ng/L in Greece and 41.2 ng/L in Spain (Alygizakis et al. 2016; María Baena-Nogueras et al. 2016). In Tunisia, our findings align with previous research by Afsa et al. (2020), which reported CFF concentrations reaching 902 µg/L in hospital effluents and 105 ng/L in seawater near site A.

In addition to CFF, the anti-epileptic drug CBZ was detected at 5.2 ng/L in a single sample from site A. Since the early 2000s, CBZ has been widely recognized as an indicator of WWTP discharge in aquatic environments (Hai et al. 2018), reinforcing the link between wastewater inputs and pharmaceutical contamination in the studied sites.

#### Sediments

Only the stimulant CFF was detected in this matrix, being ubiquitous in all analyzed samples at concentrations ranging from 4.43 to 6.84 ng/g dry weight (dw) (Table 3). The potential correlation between CFF concentrations in seawater and sediment was assessed using a Pearson correlation test, which yielded no statistically significant relationship (*p* = 0.24). While sediment concentrations remained relatively stable across sites, CFF levels in seawater varied widely (12.6–638 ng/L). This discrepancy indicates that seawater is more sensitive to episodic or localized contamination events, such as recent human activity, whereas sediment likely reflects long-term accumulation, acting as a more integrative matrix with relatively consistent concentrations.

In addition to their long-term accumulation, sediment-associated contaminants may also be subject to remobilization due to natural hydrodynamic events (e.g., storms and tidal surges) or anthropogenic activities such as dredging. These resuspension events can lead to the reintroduction of pollutants into the water column, potentially increasing their bioavailability and ecological impact. This dynamic behavior underscores the importance of considering both benthic and pelagic compartments in risk assessments and long-term monitoring strategies.

The absence of other PHACs in these sediments can be attributed to the physicochemical properties of these compounds and the environmental conditions. PHACs included in our target list present a Log Kow below 1, except for NDX (Log Kow = 1) and CBZ (Log Kow = 2.45) (Table 1). Compounds with low Log Kow values (Log Kow < 3.5) tend to be more hydrophilic and thus less likely to bind to the organic matter found in sediments, resulting in their predominant presence in the aqueous phase. Additionally, the high concentration of competing ions in seawater can further hinder the adsorption of these PHACs onto sediment particles (Zhang et al. 2020).

While no significant difference in CFF concentration ratio between sediment and seawater was observed across the three sites (*p* < 0.05), a slightly higher sediment–water ratio was noted at site B (0.08) compared to site A (0.03) and site C (0.02). To our knowledge, the current study represents the first documentation of CFF presence in sea sediments from Tunisia. Chronic exposure of aquatic organisms to CFF has been linked to oxidative stress, lipid peroxidation, neurotoxicity, alterations in energy reserves and metabolic activity, as well as impacts on reproduction and growth (González Cid 2022), sometimes leading to significant mortality rates (Baracchini et al. 2023). Hence, there is an urgent need to minimize the load of CFF into aquatic ecosystems, rationalize CFF consumption in food and beverages, and promote environmentally friendly disposal methods for caffeinated medicines.

## Environmental risk assessment

### Resistance risk quotient

The estimated RRQs revealed site-specific variations in the potential for antibiotic resistance development. At Site A, NDX, ac-SMR, OXY, and FLU posed a low risk (RRQ < 0.07); OFX posed a medium–high risk at Site A (RRQ = 0.80), suggesting a significant potential for bacterial resistance selection. Ecotoxicological assessment identified OFX as a moderate risk for fish (ERQ = 0.29) at Site C. While most PHACs exhibited ERQs < 0.01, chronic exposure to contaminant mixtures could lead to cumulative adverse effects on biota (Table 4). Although OXY has been previously identified as a high-risk compound for bacterial resistance selection (Rueanghiran et al. 2022), particularly during the summer months when higher temperatures and increased microbial activity may enhance resistance mechanisms (Bengtsson-Palme & Larsson 2016; Rueanghiran et al. 2022), its low RRQ in this study suggests that either its environmental concentrations were below critical thresholds or that local conditions influenced its bioavailability and persistence. Conversely, OFX, which exhibited the highest RRQ among the detected antibiotics, has been recognized as an emerging concern. Its recent inclusion in the European Union’s Watch List (2022) underscores the need for increased monitoring, as its environmental behavior and ecological risks are still under evaluation during a 4-year assessment period (Gomez Cortes et al. 2022). The observed variability in OFX RRQs across sites may be influenced by differences in WWTP efficiency, dilution dynamics, and sediment interactions, all of which play a role in antibiotic persistence and bioavailability. These findings highlight the site-dependent nature of antibiotic resistance risk, emphasizing the importance of targeted surveillance and mitigation strategies tailored to specific environmental conditions.

**Table 4:**
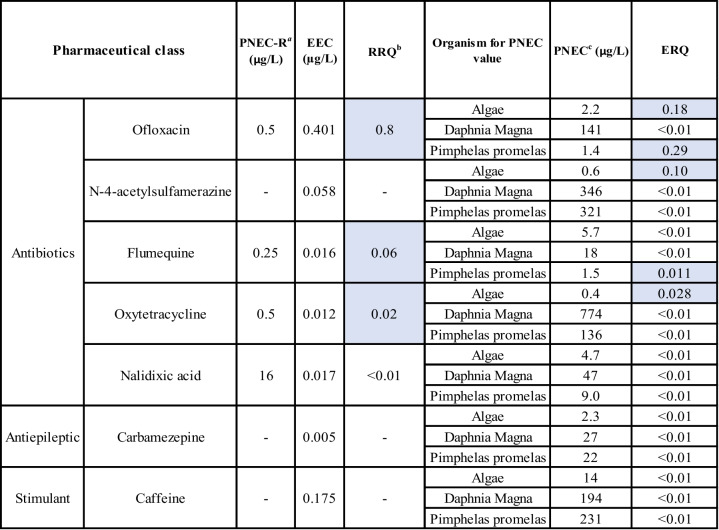
Determination of Resistance Risk Quotients (RRQs) and Ecotoxicological Risk quotient (ERQs) of detected PHACs for marine organisms. RRQs and ERQs above 0.01 are color highlighted cell in blue

^**a**^PNEC-R: estimated predicted non-effect concentration for antibiotic resistance development in the environment (µg/L), according to the NORMAN database; EEC: estimated environmental concentration of detected PHACs in collected seawater samples (µg/L) using UHPLC-MS/MS analysis (current study)

^b^RRQ: Resistant risk quotient calculated based on the PNEC-R obtained the NORMAN database

^c^PNEC: predicted no-effect concentration (the concentration below which no adverse effects are expected, derived from toxicity data and safety factors, (µg/L) according to the NORMAN database

### Ecotoxicological risk quotient

ERQs were determined for three aquatic bioindicator species representing different trophic levels: algae, crustaceans, and fish (DuLio et al. 2020). PNECs were derived from the lowest acute EC50 measured across these species (Table 4), and all values were sourced from the NORMAN database (DuLio et al. 2020).

ERQs ≥ 1 indicate a potential environmental risk, while values < 1 suggest a lower level of concern. According to ERQ values, most compounds presented low ecological risk. However, OFX showed relatively elevated ERQs, with values of 0.18 for algae and 0.29 for fish (Pimephales promelas), indicative of a potential moderate risk to primary producers and aquatic vertebrates. AcSMR also showed a notable ERQ of 0.10 for algae, suggesting moderate concern at the primary producer level. FLU and OXY, although showing ERQs well below the threshold of 1, still had ERQs of 0.011 (fish) and 0.028 (algae), respectively, which could indicate sublethal or chronic effects on aquatic organisms. The remaining compounds, including NDX, CBZ, and CFF, had ERQs < 0.01 across all tested taxa, suggesting negligible individual toxicological risk under current environmental concentrations.

Although individual contaminants may fall below critical risk thresholds, marine organisms are continuously exposed to complex mixtures of CECs. This cumulative exposure can amplify toxic effects, even at low concentrations (DuLio et al. 2020). An in vivo study (Yang et al. 2019) demonstrated dose-dependent biochemical alterations in freshwater carp (*Carassius auratus*) exposed to a mixture of OFX, sulfamethazine, and ibuprofen. These effects included significant changes in acetylcholinesterase activity, xenobiotic metabolism (7-ethoxyresorufin-O-deethylase), and antioxidant defenses (superoxide dismutase). Another study (Limbu et al. 2018) reported that chronic exposure to low concentrations of OXY and sulfamethoxazole in Nile tilapia resulted in intestinal damage, disrupted microbiota symbiosis, and oxidative stress-induced immune suppression. Therefore, while the ERQs calculated indicate a low to moderate risk from individual compounds, the potential for additive or synergistic effects from contaminant mixtures should not be overlooked. HI values calculated for cumulative risk to fish species (Table 3) were all below 1, suggesting no significant risk overall. However, the HI value for Site A in March 2019 reached 0.3, indicating a moderate level of concern when considering the combined effect of all pharmaceutical compounds present in the sample. Beyond ecological risks, antimicrobial use in aquaculture raises significant concerns regarding antibiotic resistance. The widespread application of OXY and ciprofloxacin in fish farming has been linked to increased fish growth rates, but also to the emergence and dissemination of antibiotic-resistant bacteria (Oz et al. 2014). These resistant strains can transfer to humans via the food chain or direct contact, potentially compromising infection treatment and public health.

Our results were evaluated in the context of Mediterranean regional and national water protection policies. The Barcelona Convention, formally the Convention for the Protection of the Marine Environment and the Coastal Region of the Mediterranean, adopted in 1976 and amended in 1995, establishes a legally binding framework to reduce pollution from land-based sources, including toxic, persistent, and bioaccumulative substances (Barcelona Convention for the Protection of the Mediterranean Sea Against Pollution 1976/1995). While the Convention does not currently include specific threshold values for pharmaceuticals, its land‑based sources (LBS) protocol obligates contracting parties, including Tunisia, to minimize discharges of harmful substances through national and regional action plans. At the national level, Tunisia’s water quality regulations focus on traditional pollutants (e.g., nutrients, metals, pathogens), but no specific standards for pharmaceuticals in seawater or sediments have been established (Organisation for Economic Co-operation and Development 2014). Our study, therefore, establishes a critical baseline by reporting pharmaceutical concentrations, as well as RRQ, ERQ, and HI estimates in Tunisian coastal waters, which can inform emerging regulatory efforts.

Given the Mediterranean Action Plan’s move toward achieving a “Good Environmental Status’ via the Mediterranean Pollution Assessment and Control Programme (MED POL) (United Nations Environment Programme Mediterranean Action Plan [UNEP/MAP]) and regional monitoring programs, our data provide timely insights into the occurrence of pharmaceutical pollutants and can help guide future regulatory inclusion under the LBS Protocol and related Mediterranean strategies.

## Conclusions

This study presents the first comprehensive survey of 37 pharmaceuticals in seawater and sediments along Tunisia’s central coast. Our findings confirm the presence of several PHACs, with ofloxacin reaching notorious concentrations in marine waters near Mahdia, and revealed that caffeine was consistently detected across all sampled sites. This ubiquitous occurrence of caffeine reinforces its role as an indicator of anthropogenic impact and underscores the novelty and relevance of establishing baseline for PHACs pollution.

These results also highlight the limitations of conventional wastewater treatments and points out WWTPs as point-sources of PHACs, since many of them are not fully removed during treatment. Identifying and implementing more effective treatment strategies is essential to mitigate the release of these pollutants. In this context, Tunisian environmental authorities should consider evaluating and implementing advanced treatment technologies to improve contaminants’ removal and reduce pharmaceutical discharges into coastal waters.

The detection of elevated pharmaceutical concentrations observed adjacent to aquaculture operations suggests that contribution from both on-site veterinary pharmaceuticals use and proximal WWTP effluent discharges occurs. Authorities and aquaculture managers may benefit from establishing routine monitoring of pharmaceutical residues, and their transformation products, in the surrounding environment and integrating risk assessment outcomes into farms operational planning. Such measures will help minimize ecological impacts and protect the sustainability of local aquaculture practices.

From a regulatory perspective, the current absence of pharmaceuticals discharge limits in Tunisia constitutes a challenge for controlling and mitigating environmental pollution. We advocate for the establishment of national threshold values for priority PHACs, particularly those with demonstrated ecotoxicological risk, and for the development of standardized and integrated monitoring protocols encompassing both parent compounds and their transformation products, in water and sediment. Aligning national policy with European and Mediterranean water-quality frameworks will strengthen Tunisia’s capacity to control and mitigate pharmaceutical pollution.

By providing these essential baseline data, our study also contributes to the objectives of the Barcelona Convention and supports United Nations Sustainable Development Goals 6 (“Clean Water and Sanitation”) and 14 (“Life Below Water”). Addressing pharmaceutical pollution through integrated coastal-zone management, upgraded WWTP processes, and targeted monitoring will be essential to safeguarding the long-term health and resilience of Tunisia’s marine ecosystems.

## Supplementary Information

Below is the link to the electronic supplementary material.

Supplementary file1 (DOCX 53 KB)

## Data Availability

Data will be made available on request.
