# Supplementary material for: Occurrence and risks of pharmaceuticals in Mahdia’s coastline (Tunisia): distribution, antibiotic resistance, and ecotoxicological impact
Source: Environ Sci Pollut Res Int. 2025 Jul 24;32(30):18419–33. doi: 10.1007/s11356-025-36743-3 (PMC12328540; doi:10.1007/s11356-025-36743-3)
Supplement: Supplementary file 1 — Supplementary file1 (DOCX 53 KB) [file 11356_2025_36743_MOESM1_ESM.docx]

**Occurrence and Risks of Pharmaceuticals in Mahdia’s Coastline (Tunisia): Distribution, Antibiotic Resistance, and Ecotoxicological Impact**

Ferdaws Fenni^1^, Adrià Sunyer-Caldú^2^, Hedi Ben Mansour^1^, Maria Silvia Diaz-Cruz^3^*

^1^ Research Unit of Analysis and Procedures Applied to the Environment-APAE UR17ES32, University of Tunis El Manar 1068, Tunis, Tunisia

^2^ Department of Environmental Science (ACES, Exposure & Effects), Science for Life Laboratory, Stockholm University, Stockholm 106 91, Sweden.

^3^ Consejo Superior de Investigaciones Cientificas (CSIC). Jordi Girona 18, 08034 Barcelona, Spain

*Corresponding author Email, [sdcqam@cid.csic.es](mailto:sdcqam@cid.csic.es)

**Supplementary Information**

# **Table of Content:**

[Section S1: Standards and reagents 2](#_Toc189228022)

[Section S2: Tables 5](#_Toc189228026)

# **Section S1:** Standards and reagents

Commercial standards of various pharmaceutical families, including antibiotics (fluoroquinolones, quinolones, tetracyclines, sulfonamides), lipid regulators, anti-inflammatory drugs, analgesics, β-blockers, antidepressants, antiepileptics, and stimulants were purchased with a purity >98%. Standards for nalidixic acid (NDX), oxolinic acid (OXL), tetracycline (TCY), succynil-sulfathiazole (S-STZ), N4-acetylsulfadiazidine (acSDZ), sulfadiazine (SDZ), sulfisomidine (SMD), N4- acetylsulfamerazine (acSMR), N4-acetylsulfamethazine (acSMZ), sulfamethoxazole (SMX), sulfamerazine (SMR), N4-acetylsulfamethoxazole (acSMX), sulfamethoxypyridazine (SMPZ), sulfapyridine sulfaquinoxaline (SQX), (SPY) N4-acetylsulfapyridine (acSPY), sulfathiazole (STZ), sulfadimethoxine (SDM), trimethoprim (TMP), acetaminophen, also known as paracetamol (APH), atenolol (ATL), gemfibrozil (GFZ), ketoprofen (KPF), mefenamic acid (MFA), carbamazepine (CBZ), norfluoxetine (norFXT), ofloxacin (OFX), ciprofloxacin (CFX), caffeine (CFF), ibuprofen, diclofenac (DCF), diclofenac-13C (DCF-13C) were purchased from Sigma Aldrich (Darmstadt Germany. Flumequine (FLU), N-desmethylvenlafaxine (N-desVFX), diclofenac 4-hydroxy (DCF-OH), carbamazepine 10, 11-epoxy (CBZ-E) and sulfamethazine-d4 (SMZ-d4) and acetaminophen-d4 (APH-d4) were purchased in Toronto Research Chemicals (Toronto, Canada). Oxytetracycline (OXY) and naproxen (NPX) were obtained from Honeywell Fluka (Wabash, United States). Flumequine-13C3 (FLU-13C3), trimethoprim-d3 (TMP-d3), carbamazepine-d10 (CBZ-d10), mefenamic acid-d3 (MFA-d3), caffeine-d3 (CFF-d3), ibuprofen-d3 (IBU-d3), salicylic acid-d6 (SCY-d6), diclofenac-d4 (phenyl-d4) (DCF-d4), and 5-(2,5-dimethylphenoxy)-2,2-bis(trideuteriomethyl)pentanoic acid (GMZ-d6) were from CDN isotopes (Quebec, Canada).

Water, methanol (MeOH), and acetonitrile (ACN) of MS-grade were obtained from J.T. Baker (Deventer, The Netherlands) and the nitrogen (99.995%) was supplied by Air Liquide (Barcelona, Spain). Ethanol, acetone, formic acid (FA) and ammonium acetate (AcNH4) were from Merck (Darmstadt, Germany).

Individual stock standard in addition to isotopically labelled internal standard solutions were prepared on a weight basis in MeOH at 100 mg/ L. The solutions were stored in the dark -20°C. A mixture with all the standards was weekly prepared at 20 mg/L in MeOH. Ten working solutions at concentrations between 1 ng/mL and 1 μg/mL were daily prepared by dilution of the mixture stock standard solution.

Standards stock solutions, including isotopically labelled compounds, were prepared in MeOH and stored in the dark at -20°C. A mixture standard solution was prepared weekly at 20 mg/L, while working mixture standard solutions were prepared daily.

Stock standard solutions and isotopically labelled internal standard solutions were individually prepared in MeOH at a concentration of 100 mg/L on a weight basis. All working solutions were stored in the dark at -20°C. A weekly mixture containing all standards was prepared at a concentration of 20 mg/L in MeOH. Daily working solutions, ranging from 1 ng/mL to 1 μg/mL, were prepared by diluting the stock standard solution mixture.

# **Section S2:** Tables

**Table S1.** Gradient, mobile phases, and flow used in negative ionization (ESI+) HPLC-MS/MS analysis.

| Time (min) | Flow (mL/min) | Negative ionization | |
| --- | --- | --- | --- |
|  |  | %A (MeOH ,5 mM AcNH_4_) | %B (H_2_O, 5 mMAcNH_4_) |
| 0 | 0.3 | 5 | 95 |
| 3 | 0.3 | 50 | 50 |
| 6 | 0.3 | 90 | 10 |
| 13 | 0.3 | 100 | 0 |
| 17 | 0.3 | 100 | 0 |
| 18 | 0.3 | 5 | 95 |
| 20 | 0.3 | 5 | 95 |

**Table S2.** Gradient, mobile phases, and flow used in positive ionization (ESI-) HPLC-MS/MS analysis.

| Time (min) | Flow (mL/min) | Positive ionization | |
| --- | --- | --- | --- |
|  |  | %A (MeOH, 0.1%F.A.) | %B (H_2_O, 0.1%F.A.) |
| 0 | 0.3 | 5 | 95 |
| 7 | 0.3 | 75 | 25 |
| 10 | 0.3 | 100 | 0 |
| 17 | 0.3 | 100 | 0 |
| 18 | 0.3 | 5 | 95 |
| 23 | 0.3 | 5 | 95 |

**Table S3.** HPLC-MS/MS method performance for the analysis of the target compounds. RT: chromatographic retention time; SRM1: MS/MS transition 1 (quantification); SRM2: MS/MS transition 2 (confirmation); LOD seawater: method limit of detection (ng/L); LOQ seawater: method limit of quantification (ng/L); LOD sediment: method limit of detection (ng/g dw); LOQ sediment: method limit of quantification (ng/g dw); r2: Linearity. Reported values were originally provided in (Fenni et al., 2022), (Gago-Ferrero et al., 2011), and (Díaz-Cruz & Barceló, 2005).

| **Name (Acronym)** | **Internal standard** | **RT (min)** | **SRM1** | **SRM2** | **LOD**  **seawater** | **LOQ seawater** | **LOD sediment** | **LOQ sediment** | **r^2^**  **(seawater)** | **r^2^ (sediment)** |
| --- | --- | --- | --- | --- | --- | --- | --- | --- | --- | --- |
| Flumequine (FLU) | Flumequine-^13^C | 6.7 | 262>244 | 262>107 | 0.48 | 1.60 | 0.39 | 1.31 | 0.9977 | 0.9983 |
| Ofloxacin (OFX) | Sulfamethazine-d_4_ | 4.5 | 362>318 | 362>261 | 2.53 | 8.44 | 0.36 | 1.2 | 0.9401 | 0.9986 |
| Ciprofloxacin (CFX) | Triethoprim-d_3_ | 4.2 | 332>288 | 332>231 | 0.58 | 1.93 | 0.74 | 2.47 | 0.9966 | 0.994 |
| Nalidixic acid (NDX) | Flumequine-^13^C | 6.6 | 233>215 | 233>187 | 0.57 | 1.89 | 0.35 | 1.16 | 0.9967 | 0.9987 |
| Oxolinic acid (OXL) | Flumequine-^13^C | 5.8 | 262>244 | 262>216 | 0.30 | 1.00 | 0.4 | 1.33 | 0.9991 | 0.9982 |
| Tetracycline (TCY) | Triethoprim-d_3_ | 4.1 | 445>410 | 445>427 | 1.94 | 6.46 | 1.06 | 3.53 | 0.9635 | 0.9878 |
| Oxytetracycline (OXY) | Triethoprim-d_3_ | 3.9 | 461>426 | 461>443 | 0.52 | 1.72 | 1.02 | 3.42 | 0.9973 | 0.9886 |
| Succinyl Sulfathiazole (S-STZ) | Sulfamethazine-d_4_ | 4.3 | 356>256 | 356>192 | 0.20 | 0.67 | 1.07 | 3.57 | 0.9996 | 0.9876 |
| Sulfadiazine (SDZ) | Sulfamethazine-d_4_ | 4.4 | 251>156 | 251>108 | 0.69 | 2.29 | 1.00 | 3.33 | 0.9952 | 0.9892 |
| N4-acetylSulfadiazidine  (ac-SDZ) | Sulfamethazine-d_4_ | 4.6 | 307>134 | 307>110 | 0.55 | 1.83 | 1.10 | 3.68 | 0.9969 | 0.9868 |
| Sulfamerazine (SMR) | Sulfamethazine-d_4_ | 4.4 | 265>156 | 256>92 | 0.78 | 2.59 | 1.12 | 3.75 | 0.9939 | 0.9863 |
| N4-acetylSulfamerazine(ac-SMR) | Sulfamethazine-d_4_ | 4.6 | 307>134 | 307>110 | 0.81 | 2.70 | 0.84 | 2.79 | 0.9934 | 0.9924 |
| N4-acetylSulfamethiazine(ac-SMZ) | Sulfamethazine-d_4_ | 4.7 | 321>134 | 321>124 | 0.88 | 2.94 | 1.14 | 3.78 | 0.9921 | 0.986 |
| Sulfamethoxazole (SMX) | Sulfamethazine-d_4_ | 5.6 | 254>108 | 254>156 | 0.65 | 2.16 | 1.02 | 3.41 | 0.9957 | 0.9886 |
| N4-acetylSulfamethoxazole (ac-SMX) | Sulfamethazine-d_4_ | 5.8 | 296>198 | 296>134 | 0.50 | 1.66 | 1.59 | 5.30 | 0.9975 | 0.9731 |
| Sulfamethoxypyridazine (SMPZ) | Sulfamethazine-d_4_ | 4.7 | 281>126 | 281>156 | 0.62 | 2.07 | 0.89 | 2.96 | 0.9961 | 0.9914 |
| Sulfapyridine (SPY) | Sulfamethazine-d_4_ | 4.2 | 250>156 | 250>92 | 0.94 | 3.14 | 0.97 | 3.22 | 0.991 | 0.9898 |
| N4-Sulfapyridine (ac-SPY) | Sulfamethazine-d_4_ | 4.5 | 292>134 | 292>198 | 0.92 | 3.06 | 1.27 | 4.24 | 0.9915 | 0.9826 |
| Sulfaquinoxaline (SQX) | Sulfamethazine-d_4_ | 6 | 301>156 | 301>198 | 0.73 | 2.43 | 1.04 | 3.47 | 0.9946 | 0.9882 |
| Sulfathiazole (STZ) | Sulfamethazine-d_4_ | 4.1 | 256>156 | 256>92 | 0.75 | 2.50 | 1.03 | 3.44 | 0.9943 | 0.9884 |
| Sulfisomidine (SMD) | Sulfamethazine-d_4_ | 4.6 | 279>124 | 279>186 | 0.69 | 2.31 | 0.72 | 2.42 | 0.9951 | 0.9942 |
| Sulfadimethoxine (SDM) | Sulfamethazine-d_4_ | 6.1 | 311>156 | 311>92 | 0.60 | 2.01 | 0.92 | 3.07 | 0.9963 | 0.9907 |
| Trimethoprim (TMP) | Trimethoprim-d_3_ | 3.9 | 291>230 | 291>261 | 0.81 | 2.68 | 0.90 | 2.98 | 0.9934 | 0.9912 |
| Gemfibrozil (GFZ) | Gemfibrozil-d_6_ | 9.2 | 251>233 | 251>129 | 0.45 | 1.51 | 1.08 | 3.61 | 0.9979 | 0.9873 |
| Mefamic acid (MFA) | Mefamic acid -d_3_ | 9.2 | 242>224 | 242>209 | 0.37 | 1.23 | 1.81 | 6.05 | 0.9986 | 0.9654 |
| Naproxen (NPX) | Flumequine-^13^C | 4.3 | 231>185 | 231>115 | 0.77 | 2.57 | 0.41 | 1.36 | 0.994 | 0.9982 |
| Iburofen (IBU) | Ibuprofen-d_3_ | 5.3 | 205>159 | 205>161 | 0.55 | 1.83 | 0.63 | 2.10 | 0.9969 | 0.9956 |
| Ketoprofen (KPF) | BP-3d_5_ | 7.3 | 255>105 | 255<209 | 0.55 | 1.83 | 2.77 | 9.22 | 0.9969 | 0.9247 |
| Diclofenac (DCF) | diclofenac-d_~~4~~_(phenyl-d_4_) | 4.9 | 295>251 | 295>215 | 1.14 | 3.81 | 3.26 | 10.86 | 0.9869 | 0.8998 |
| Paracetamol (APH) | Acetaminophen-d_4_ | 3.6 | 152>110 | 152>65 | 0.58 | 1.92 | 0.99 | 3.31 | 0.9966 | 0.9893 |
| Carbamazepine (CBZ) | Carbamezepine-d_10_ | 6.5 | 237>194 | 237>192 | 0.39 | 1.31 | 0.73 | 2.43 | 0.9984 | 0.9942 |
| Epoxycarbamazepine-10,11 (CBZ-E) | Carbamezepine-d_10_ | 5.7 | 253>180 | 253>151 | 0.81 | 2.71 | 0.95 | 3.18 | 0.9933 | 0.9901 |
| Atenol (ATL) | Trimethoprim-d_3_ | 3.1 | 267>116 | 267>133 | 0.33 | 1.11 | 0.83 | 2.78 | 0.9989 | 0.9924 |
| Nofloxetine (NorFXT) | Trimethoprim-d_3_ | 5.6 | 296>134 | 296>59 | 0.78 | 2.60 | 1.67 | 5.57 | 0.9938 | 0.9705 |
| N-Desmethylvenlafaxine (N-desVFX) | Trimethoprim-d_3_ | 4.4 | 264>58 | 264>133 | 0.90 | 3.00 | 0.85 | 2.84 | 0.9918 | 0.9921 |
| Caffeine (CFF) | Caffeine-d_3_ | 4 | 195>109 | 195>138 | 0.63 | 2.08 | 1.02 | 3.40 | 0.996 | 0.9887 |

**Table S4.** Recovery rates and associated standard deviation (RSD) for water and sediment samples.

| **Name (Acronym)** | **Internal standard** | **5xLOQ Recovery water (R% ± RSD %)** | **10xLOQ Recovery water (R% ± RSD %)** | **5xLOQ Recovery sediment (R% ± RSD %)** | **10xLOQ Recovery sediment (R% ± RSD %)** |
| --- | --- | --- | --- | --- | --- |
|  |  |  |  |  |  |
| Flumequine (FLU) | Flumequine-^13^C | 101.7 ± 7.6 | 105.7 ± 5 | 75.6 ± 10.5 | 60.8 ± 9.7 |
| Ofloxacin (OFX) | Sulfamethazine-d_4_ | 118.5 ± 2.3 | 103.1 ± 7.6 | 130.5 ± 26.6 | 124.9 ± 12.4 |
| Ciprofloxacin (CFX) | Triethoprim-d_3_ | 100.1 ± 25.4 | 100.6 ± 10.1 | 82.2 ± 10.0 | 72.2 ± 9.0 |
| Nalidixic acid (NDX) | Flumequine-^13^C | 115.9 ± 15.9 | 96.4 ± 11.1 | 123.3 ± 9.6 | 75.6 ± 11.5 |
| Oxolinic acid (OXL) | Flumequine-^13^C | 106.1 ± 2.6 | 100.4 ± 10 | 62.2 ± 24.8 | 75.0 ± 7.7 |
| Tetracycline (TCY) | Triethoprim-d_3_ | 91.2 ± 10.8 | 86.3 ± 14.7 | 74.8 ± 12.8 | 81.2 ± 9.8 |
| Oxytetracycline (OXY) | Triethoprim-d_3_ | 102.9 ± 13.4 | 102.7 ± 4.5 | 79.4 ± 22.2 | 66.1 ± 8.2 |
| Succinyl Sulfathiazole (S-STZ) | Sulfamethazine-d_4_ | 103.2 ± 0.6 | 136.3 ± 3.2 | 79.9 ± 4.2 | 122.8 ± 4.7 |
| Sulfadiazine (SDZ) | Sulfamethazine-d_4_ | 70.6 ± 2.8 | 97 ± 3.6 | 102.9 ± 29.2 | 121.7 ± 20.5 |
| N4-acetylSulfadiazidine | Sulfamethazine-d_4_ | 95.9 ± 1.5 | 96.9 ± 10.8 | 124.7 ± 6.1 | 85.6 ± 13.8 |
| (ac-SDZ) |  |  |  |  |  |
| Sulfamerazine (SMR) | Sulfamethazine-d_4_ | 89.3 ± 3.2 | 99.4 ± 4.6 | 122.4 ± 23.6 | 81.9 ± 24.4 |
| N4-acetylSulfamerazine(ac-SMR) | Sulfamethazine-d_4_ | 93 ± 1.2 | 105.3 ± 3.2 | 69.4 ± 5.3 | 99.9 ± 22.0 |
| N4-acetylSulfamethiazine(ac-SMZ) | Sulfamethazine-d_4_ | 97.6 ± 3.8 | 101.6 ± 7.3 | 89.5 ± 3.7 | 129.7 ± 5.8 |
| Sulfamethoxazole (SMX) | Sulfamethazine-d_4_ | 96 ± 10.7 | 113.5 ± 23.5 | 78.3 ± 9.1 | 131.0 ± 5.8 |
| N4-acetylSulfamethoxazole (ac-SMX) | Sulfamethazine-d_4_ | 101.9 ± 10.8 | 104.5 ± 13.3 | 70.0 ± 6.6 | 105.4 ± 7.0 |
| Sulfamethoxypyridazine (SMPZ) | Sulfamethazine-d_4_ | 108.3 ± 24.9 | 99.3 ± 19.3 | 88.0 ± 3.0 | 122.3 ± 5.4 |
| Sulfapyridine (SPY) | Sulfamethazine-d_4_ | 100.9 ± 10.3 | 112.7 ± 6.8 | 74.4 ± 3.3 | 111.6 ± 14.9 |
| N4-Sulfapyridine (ac-SPY) | Sulfamethazine-d_4_ | 89.2 ± 8.8 | 104.5 ± 16.3 | 49.3 ± 6.8 | 71.3 ± 0.1 |
| Sulfaquinoxaline (SQX) | Sulfamethazine-d_4_ | 102.3 ± 10.5 | 99.2 ± 4.1 | 97.6 ± 11.1 | 105.6 ± 20.0 |
| Sulfathiazole (STZ) | Sulfamethazine-d_4_ | 96.2 ± 14.9 | 127.7 ± 10.1 | 72.6 ± 26.2 | 115.7 ± 8.7 |
| Sulfisomidine (SMD) | Sulfamethazine-d_4_ | 102.7 ± 5.3 | 111.6 ± 2 | 74.4 ± 26.3 | 65.7 ± 3.8 |
| Sulfadimethoxine (SDM) | Sulfamethazine-d_4_ | 115.9 ± 12.5 | 117.2 ± 18.7 | 103.7 ± 4.3 | 91.8 ± 4.3 |
| Trimethoprim (TMP) | Trimethoprim-d_3_ | 99.7 ± 13.3 | 106 ± 8.9 | 78.5 ± 16.7 | 98.8 ± 21.5 |
| Gemfibrozil (GFZ) | Gemfibrozil-d_6_ | 104.3 ± 10.5 | 101 ± 13.1 | 68.3 ± 15.3 | 68.5 ± 5.8 |
| Mefamic acid (MFA) | Mefamic acid -d_3_ | 114 ± 19.2 | 92.6 ± 1 | 95.8 ± 9.3 | 123.1 ± 25.3 |
| Naproxen (NPX) | Flumequine-^13^C | 106.1 ± 5.5 | 107.2 ± 9.1 | 65.3 ± 5.4 | 112.0 ± 18.9 |
| Iburofen (IBU) | Ibuprofen-d_3_ | 77.8 ± 14.2 | 73.1 ± 11.6 | 78.9 ± 17.5 | 111.8 ± 17.9 |
| Ketoprofen (KPF) | BP-3d_5_ | 112.2 ± 2.1 | 104.9 ± 7.7 | 74.0 ± 10.9 | 106.5 ± 11.0 |
| Diclofenac (DCF) | diclofenac-d_~~4~~_(phenyl-d_4_) | 112.5 ± 14.1 | 97.3 ± 2.9 | 111.5 ± 25.7 | 59.4 ± 17.9 |
| Paracetamol (APH) | Acetaminophen-d_4_ | 105.3 ± 3.4 | 104.8 ± 8 | 74.8 ± 24.2 | 102.6 ± 27.2 |
| Carbamazepine (CBZ) | Carbamezepine-d_10_ | 110.1 ± 13 | 101.6 ± 6 | 103.6 ± 12.8 | 103.2 ± 9.4 |
| Epoxycarbamazepine-10,11 (CBZ-E) | Carbamezepine-d_10_ | 112.5 ± 7.2 | 102.5 ± 4.3 | 73.3 ± 13.5 | 109.3 ± 2.1 |
| Atenol (ATL) | Trimethoprim-d_3_ | 90.9 ± 4.3 | 89.9 ± 16.5 | 92.0 ± 1.7 | 138.1 ± 1.5 |
| Nofloxetine (NorFXT) | Trimethoprim-d_3_ | 101.8 ± 6.9 | 102.9 ± 7.3 | 93.4 ± 0.3 | 70.1 ± 14.7 |
| N-Desmethylvenlafaxine (N-desVFX) | Trimethoprim-d_3_ | 99 ± 5.5 | 116.3 ± 10.6 | 131.4 ± 16.4 | 66.3 ± 13.3 |
| Caffeine (CFF) | Caffeine-d_3_ | 95.9 ± 5 | 108.8 ± 17.8 | 115.3 ± 0.4 | 122.3 ± 13.7 |

# **References:**

Díaz-Cruz, M. S., & Barceló, D. (2005). LC–MS2 trace analysis of antimicrobials in water, sediment and soil. *TrAC Trends in Analytical Chemistry*, *24*(7), 645–657. https://doi.org/10.1016/j.trac.2005.05.005

Fenni, F., Sunyer-Caldú, A., Ben Mansour, H., & Diaz-Cruz, M. S. (2022). Contaminants of emerging concern in marine areas: First evidence of UV filters and paraben preservatives in seawater and sediment on the eastern coast of Tunisia. *Environmental Pollution*, *309*, 119749. https://doi.org/10.1016/j.envpol.2022.119749

Gago-Ferrero, P., Díaz-Cruz, M. S., & Barceló, D. (2011). Fast pressurized liquid extraction with in-cell purification and analysis by liquid chromatography tandem mass spectrometry for the determination of UV filters and their degradation products in sediments. *Analytical and Bioanalytical Chemistry*, *400*(7), 2195–2204. https://doi.org/10.1007/s00216-011-4951-1
